# Trend changing to the access category antibiotic usage after digital antimicrobial stewardship tool E-RASPRO 9 months implementation in an Indonesian hospital

**DOI:** 10.1017/ash.2025.107

**Published:** 2025-09-03

**Authors:** Hadianti Adlani, Aziza Ariyani, Ronald Irwanto Natadidjaja, Anti Dharmayanti

**Affiliations:** 1 Indonesian Society of Infection Control (INASIC) Branch Banten; 2 RASPRO Indonesia *Study Group*; 3 Faculty of Medicine Universitas Trisakti

**Keywords:** digital antimicrobial stewardship, Define Daily Dose, ACCESS, WATCH

## Abstract

**Background:** Antimicrobial Stewardship Program (ASP) is a global issue. World Health Organization (WHO) stated, there are 3 categories of antimicrobial: ACCESS, WATCH, and RESERVE. e-RASPRO as a digital ASP may alter antibiotic prescribing pattern by prioritizing ACCESS category as suggested by WHO. **Methods:** This manuscript was a ward retrospective survey data of 9 months Define Daily Dose (DDD) average before-after implementing the electronic-RASPRO (e-RASPRO) on ACCESS & WATCH antibiotic. **Results:** Number of inpatients 9 months before-after e-RASPRO implementation were 7,754 and 6,794. Within 9 months after implementing e-RASPRO there was a trend of antibiotic prescription shifting from WATCH category antibiotic to ACCESS category antibiotic. There was a trend of reduced Define Daily Dose (DDD) average of WATCH category antibiotic. 24.82% of 3^rd^ generation Cephalosporin, 33.20% of Quinolones, 14.76% of Carbapenems and 100% of Piperacillin Tazobactam DDD average were reduced. While, in ACCESS Category Antibiotic, there were an elevation of Penicillin and Aminoglycosides DDD average up to 528.66% and 137.66%. **Conclusion:** **T** here are trend changing of DDD average from WATCH to ACCESS category antibiotic following the 9 months implementation of e-RASPRO. We need further study to judge the effectiveness of e- RASPRO as a digital ASP tools.

